# Supplementary material for: Patients’ experiences with musculoskeletal spinal pain: A qualitative systematic review protocol
Source: PLoS One. 2024 Aug 8;19(8):e0306993. doi: 10.1371/journal.pone.0306993 (PMC11309383; doi:10.1371/journal.pone.0306993)
Supplement: S1 Table — (DOCX) [file pone.0306993.s001.docx]

S1 Table. Search strategy Medline (OVID).

| **#** | **Searches** |
| --- | --- |
| 1 | exp Empirical Research/ or Interviews as Topic/ or Personal Narratives as Topic/ or Focus Groups/ or exp Narration/ or Nursing Methodology Research/ or Narrative Medicine/ |
| 2 | (Interview or Personal Narrative).pt. |
| 3 | interview*.ti,ab,kf. |
| 4 | qualitative.ti,ab,kf,jw. |
| 5 | (theme* or thematic).ti,ab,kf. |
| 6 | ethnological research.ti,ab,kf. |
| 7 | ethnograph*.ti,ab,kf. |
| 8 | ethnomedicine.ti,ab,kf. |
| 9 | ethnonursing.ti,ab,kf. |
| 10 | phenomenol*.ti,ab,kf. |
| 11 | (grounded adj (theor* or study or studies or research or analys?s)).ti,ab,kf. |
| 12 | life stor*.ti,ab,kf. |
| 13 | (emic or etic or hermeneutic* or heuristic* or semiotic*).ti,ab,kf. |
| 14 | (data adj1 saturat$).ti,ab,kf. |
| 15 | participant observ*.ti,ab,kf. |
| 16 | (social construct* or postmodern* or post-structural* or post structural* or poststructural* or post modern* or post-modern*).ti,ab,kf. |
| 17 | (action research or cooperative inquir* or co operative inquir* or co-operative inquir*).ti,ab,kf. |
| 18 | (humanistic or existential or experiential or paradigm*).ti,ab,kf. |
| 19 | (field adj (study or studies or research or work)).ti,ab,kf. |
| 20 | (human science or social science).ti,ab,kf. |
| 21 | biographical method.ti,ab,kf. |
| 22 | theoretical sampl*.ti,ab,kf. |
| 23 | ((purpos* adj4 sampl*) or (focus adj group*)).ti,ab,kf. |
| 24 | (open-ended or narrative* or textual or texts or semi-structured).ti,ab,kf. |
| 25 | (life world* or life-world* or conversation analys?s or personal experience* or theoretical saturation).ti,ab,kf. |
| 26 | ((lived or life) adj experience*).ti,ab,kf. |
| 27 | cluster sampl*.ti,ab,kf. |
| 28 | observational method*.ti,ab,kf. |
| 29 | content analysis.ti,ab,kf. |
| 30 | (constant adj (comparative or comparison)).ti,ab,kf. |
| 31 | ((discourse* or discurs*) adj3 analys?s).ti,ab,kf. |
| 32 | (heidegger* or colaizzi* or spiegelberg* or merleau* or husserl* or foucault* or ricoeur or glaser*).ti,ab,kf. |
| 33 | (van adj manen*).ti,ab,kf. |
| 34 | (van adj kaam*).ti,ab,kf. |
| 35 | (corbin* adj2 strauss*).ti,ab,kf. |
| 36 | 1 or 2 or 3 or 4 or 5 or 6 or 7 or 8 or 9 or 10 or 11 or 12 or 13 or 14 or 15 or 16 or 17 or 18 or 19 or 20 or 21 or 22 or 23 or 24 or 25 or 26 or 27 or 28 or 29 or 30 or 31 or 32 or 33 or 34 or 35 |
| 37 | low back pain.mp. or exp Low Back Pain/ |
| 38 | sciatica.mp. or exp Sciatica/ |
| 39 | radiculopathy.mp. or exp Radiculopathy/ |
| 40 | ((lumbar or lumbosacral or lumbo-sacral or back) adj5 (pain* or ache* or aching)).ti,ab,kf. |
| 41 | (backache* or lumbago or sciatica).ti,ab,kf. |
| 42 | (radiculopathy or radiculitis or radicular pain*).ti,ab,kf. |
| 43 | (nerve root* adj5 (pain* or avulsion or compress* or disorder* or pinch* or inflam* or imping' or irritat* or entrap* or trap*)).ti,ab,kf. |
| 44 | 42 or 43 |
| 45 | (back* or lumbosacral or lumbo-sacral or lumbar).ti,ab,kf. |
| 46 | 44 and 45 |
| 47 | 37 or 38 or 39 or 40 or 41 or 46 |
| 48 | Neck Pain.mp. or exp Neck Pain/ |
| 49 | exp Brachial Plexus Neuropathies/ |
| 50 | cervical pain.ti,ab,kf. |
| 51 | neckache.ti,ab,kf. |
| 52 | cervicodynia.ti,ab,kf. |
| 53 | cervicalgia.ti,ab,kf. |
| 54 | brachialgia.ti,ab,kf. |
| 55 | brachial neuritis.ti,ab,kf. |
| 56 | brachial neuralgia.ti,ab,kf. |
| 57 | brachial plexus neuropath*.ti,ab,kf. |
| 58 | brachial plexus neuritis.ti,ab,kf. |
| 59 | exp brachial plexus neuropathies/ or exp brachial plexus neuritis/ |
| 60 | cervico brachial neuralgia.ti,ab. |
| 61 | cervicobrachial neuralgia.ti,ab. |
| 62 | (monoradicul* or monoradicl*).tw. |
| 63 | 48 or 49 or 50 or 51 or 52 or 53 or 54 or 55 or 56 or 57 or 58 or 59 or 60 or 61 or 62 |
| 64 | exp Thoracic Vertebrae/ or thoracic spine.ti,ab,kf. |
| 65 | dorsal spine.ti,ab,kf. |
| 66 | (mid* back or upper back or thoracolumbar or cervicothoracic).ti,ab,kf. |
| 67 | pain.mp. or exp Pain/ |
| 68 | 64 or 65 or 66 |
| 69 | 67 and 68 |
| 70 | 47 or 63 or 69 |
| 71 | ("Experiences" or "Lived experiences" or "Narratives" or "Personal narratives" or "Perceptions" or "Stories" or "Viewpoints" or "Insights" or "Personal accounts" or "Qualitative experiences" or "Subjective experiences" or "Individual experiences" or "Journey" or "Testimonies" or "Health experiences" or "Experiential accounts" or "Personal reflections" or "Feedback" or "Life experiences" or "Satisfaction" or "Perspectives" or "Personal experiences" or "First-hand experiences" or "Reported outcomes" or "Experiential narratives" or "Personal perceptions" or "Qualitative feedback" or "Voices" or "Personal journeys" or "Engagement experiences" or "Emotional experiences" or "Psychological experiences" or "Coping experiences" or "Health journey narratives" or "Treatment experiences" or "Care experiences" or "Disease impact narratives" or "Wellness journeys" or "Centered experiences" or "Healthcare experiences").ti,ab,kf. |
| 72 | 36 or 71 |
| 73 | 70 and 72 |

pt: Publication Type

ti.ab.kf.: Title/Abstract/Keyword Field

exp: Explode (includes all subheadings)

adj: Adjacency (proximity search)

mp: Multiple Posting

tw: Text Word
