# Supplementary material for: Patients’ experiences with musculoskeletal spinal pain: A qualitative systematic review protocol
Source: PLoS One. 2024 Aug 8;19(8):e0306993. doi: 10.1371/journal.pone.0306993 (PMC11309383; doi:10.1371/journal.pone.0306993)
Supplement: S2 Table — (DOCX) [file pone.0306993.s002.docx]

**S2 Table. Data extraction form.**

**Study title:**

| Study characteristics. |  |
| --- | --- |
| Publication year. | Year in which the study was published. |
| Geographic location. | The location where the study was conducted. |
| Sample characteristics. | Information about the study participants (e.g., size, demographics), and inclusion/exclusion criteria. |
| Size. |  |
| Participant demographics. |  |
| Inclusion/exclusion criteria. |  |
| Study aims. | The primary objectives and goals of the study. |
| Study design (methodological underpinnings). | Methodological approach and underpinnings of the study (e.g., grounded theory, phenomenology). |
| Data collection methods. | How data was collected in the study (e.g., interviews, surveys, observations). |
| Key findings and illustrations. | The main findings of the study, including authors’ verbatim analytical interpretations, and any supporting illustrations (e.g., quotes, narratives). |

**Authors:**
